# Bilateral Theta-Burst TMS to Influence Global Gestalt Perception

**DOI:** 10.1371/journal.pone.0047820

**Published:** 2012-10-26

**Authors:** Bernd Ritzinger, Elisabeth Huberle, Hans-Otto Karnath

**Affiliations:** 1 Division of Neuropsychology, Center of Neurology, Hertie-Institute for Clinical Brain Research, University of Tübingen, Tübingen, Germany; 2 Neurology and Neurorehabilitation Center, Luzerner Kantonsspital, Lucerne, Switzerland; 3 Department of Psychology, University of South Carolina, Columbia, South Carolina, United States of America; Indiana University, United States of America

## Abstract

While early and higher visual areas along the ventral visual pathway in the inferotemporal cortex are critical for the recognition of individual objects, the neural representation of human perception of complex global visual scenes remains under debate. Stroke patients with a selective deficit in the perception of a complex global Gestalt with intact recognition of individual objects – a deficit termed simultanagnosia – greatly helped to study this question. Interestingly, simultanagnosia typically results from bilateral lesions of the temporo-parietal junction (TPJ). The present study aimed to verify the relevance of this area for human global Gestalt perception. We applied continuous theta-burst TMS either unilaterally (left or right) or bilateral simultaneously over TPJ. Healthy subjects were presented with hierarchically organized visual stimuli that allowed parametrical degrading of the object at the global level. Identification of the global Gestalt was significantly modulated only for the bilateral TPJ stimulation condition. Our results strengthen the view that global Gestalt perception in the human brain involves TPJ and is co-dependent on both hemispheres.

## Introduction

Theories of higher-level visual perception differentiate between the perception of individual elements at the local level and of global objects, for which the information of many local components are perceptually grouped to form a global Gestalt [1]–[3]. Investigations on how local and global processing are implemented in the brain have put a major focus on the inferotemporal cortex (IT). IT cortex has been identified to be critically involved in the recognition of individual objects [4]–[9]. It has been proposed that visual information – in a bottom-up manner – is passed from early visual areas, which are involved in the analysis of local features, to higher order areas, where a coherent global percept results from grouping of local elements [10]–[12]. In addition, recent findings have argued for a substantial role of top-down processes, where a fast and rough estimation of the global Gestalt is related to local processing circuits in the IT cortex by narrowing down the variety of possible lower level outcomes [13], [14]. Neurophysiological evidence has been reported that both local and global information of hierarchical stimuli is processed in the same cells of monkey IT cortex [15]–[17]. Human fMRI studies in healthy subjects have demonstrated an involvement of early visual areas V1 and V2 as well as area V4 and occipito-temporal areas in global Gestalt perception [18]. In line with the above findings are observations in neurological patients suffering from bilateral lesions of IT cortex and visual form agnosia [19]–[21]. These patients have lost the ability to discriminate between simple geometric shapes and orientation as well as to recognise objects, despite the fact that basic visual abilities like the analysis of contrast, colour, or motion remain largely intact.

In contrast to a deficit in shape, orientation or object recognition, the critical neural correlate of global Gestalt perception appears to be located more dorsally and is clearly distinct from IT cortex. Patients with bilateral lesions in the temporo-parietal junction (TPJ) area [22]–[31] are not capable to perceive global scenes while local recognition of single objects remains intact – a deficit termed ‘simultanagnosia’ [32]–[34]. Such patients are not able to perceive more than a single object at a time and fail to perceptually group the single objects of a complex scene to a global Gestalt [26], [29], [30], [32], [35], [36]. Only few patients were reported with unilateral lesions of the TPJ and simultanagnosia [37]–[38].

A recent event-related fMRI study with a simultanagnostic patient allowed to further narrow down the region involved in global Gestalt perception [39]. This patient exhibited incomplete simultanagnosia, which offered the possibility to post hoc select and directly contrast brain activation in trials of successful global recognition with trials of global recognition failure. Bilateral clusters of activity in the inferior parietal lobule and ventral precuneus correlated with the ability to recognize the global scene. In addition, recent fMRI data have revealed activation of the TPJ area and the precuneus bilaterally with global gestalt perception in healthy subjects [40].

Neuroimaging studies as well as neuropsychological and neurophysiological work in humans also suggested a differentiation of local and global processing between hemispheres. Several studies reported a right hemispheric predominance for global Gestalt perception together with a left hemispheric predominance for local perception [41]–[45]. Also, an influence of perceptual saliency and spatial frequency on determining the cerebral organization of global versus local processing has been observed [46].

In summary, previous data favor a bilateral representation of global Gestalt perception in humans over a unilateral implementation. To address the question of unilateral (right hemisphere) versus bilateral representation of global Gestalt perception and to verify the relevance of the TPJ for grouping of local objects into a global Gestalt, we applied continuous theta-burst TMS (cTBS) in healthy subjects either unilaterally (left or right) or bilateral simultaneously over TPJ. cTBS is known to produce suppressing effects on motor-evoked potentials (MEP) after application over motor cortex [47], [48]. We thus hypothesized comparable effects in the stimulated areas with a decrease in performance – in parallel to patients with simultanagnosia.

## Methods

### Subjects

Fifteen healthy volunteers participated in this study (9 females, 6 males), with a median age of 26 years (range 20–35). All subjects had normal or corrected to normal vision and were right-handed. Experiments followed the safety guidelines for rTMS/cTBS protocols established by Wassermann [49] and Rossi et al. [50] and were approved by the local ethics committee. All subjects gave their written informed consent to participate in this study and were instructed about possible side effects during or following TMS stimulation. Four of the fifteen subjects reported mild headache.

### Stimuli and Presentation Procedure

**Figure 1:**
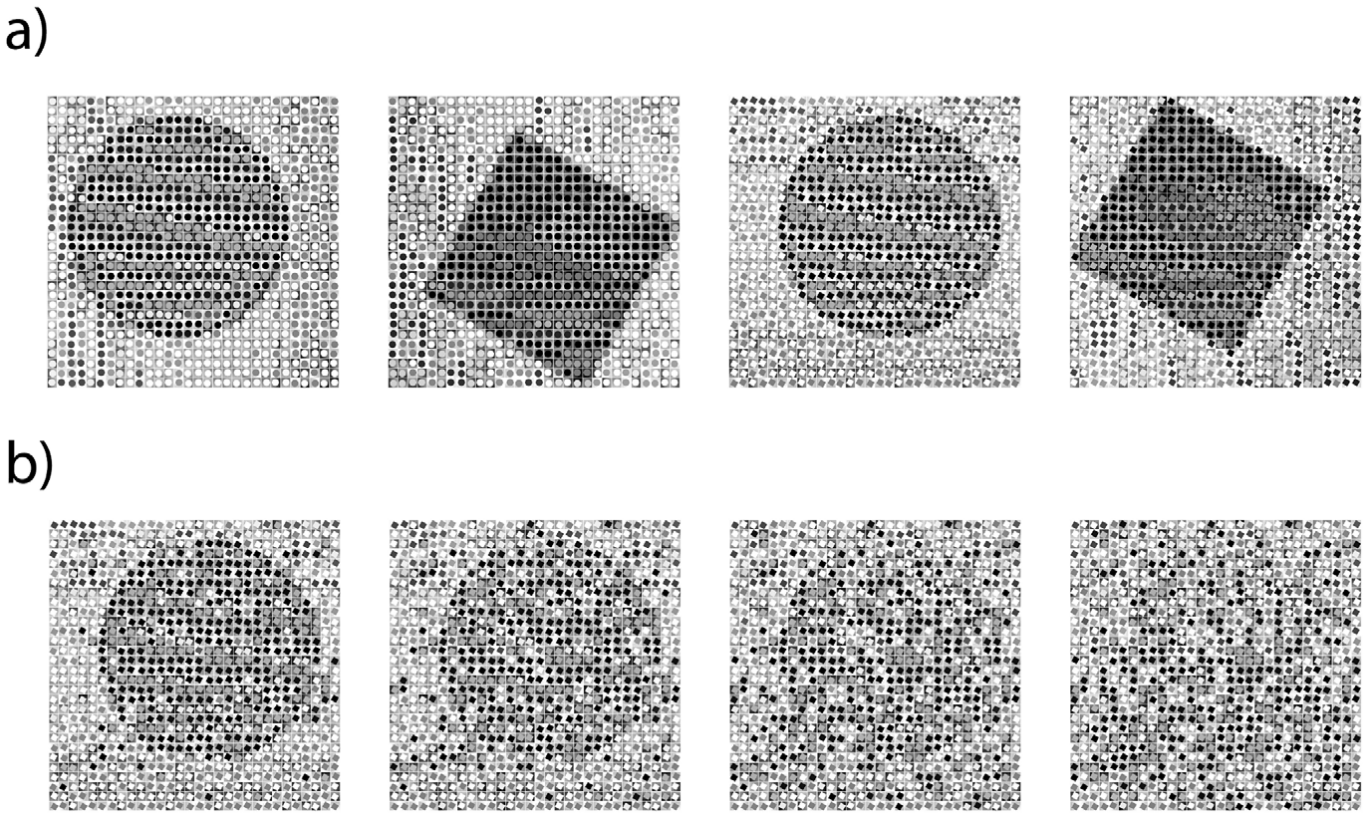
Experimental stimuli. (a) The four stimulus conditions used in the experiment. (b) Example of scrambled stimuli: A global circle with local squares presented with 20%, 40%, 60% and 80% scrambling rate. The global information is distorted, while the local contents are unchanged.

Hierarchical stimuli were presented on a PC monitor on a medium gray background (RGB-Value = 7e7e7e) at a viewing distance of 50 cm in daylight condition. The stimuli consisted of 30×30 local gray-scale images of circles or squares with different contrasts, which were arranged to form global circles or squares (visual angle of stimulus at global level: 7.78°×7.78°, local elements: 0.26°×0.26°; cf. Fig. 1a). This procedure resulted in four conditions: global circle/local circle, global circle/local square, global square/local circle, and global square/local square. In order to manipulate global Gestalt perception and determine the subjects’ limits, the global objects were scrambled by exchanging the elements at the local level by 20%, 40%, 60% and 80%. This procedure allowed disturbed global Gestalt perception in the context of intact local perception (cf. Fig. 1b) and was used in a similar fashion in a previous study [40].

For each TMS application site (see below) 96 trials of visual stimulus presentation were used in an order which was balanced for the degree of scrambling as well as the object at the local and global level. Each trial lasted for 3000 msec, resulting in an experimental time for each stimulation site of 288 sec (see also ‘Stimulation locations’ below). Following an initial fixation period of 500 ms (+/−5 ms of random jitter) at the beginning of each trial with a central fixation point on the presentation screen, the stimulus appeared for 300 ms and was replaced by a final fixation period for the rest of the trial during which the subject’s response was coded.

Subjects were engaged in a two-alternative forced choice task and instructed to fixate the fixation point on the presentation screen throughout the experiment. The task required the identification of the object at the global level of the stimuli (circle or square). The response and reaction time were coded by the subject’s key press on a standard computer keyboard.

In five subjects, we also run a control experiment in which we asked to identify the object at the local level (circle or square), using all four unilateral and bilateral simultaneous stimulation conditions as described below. The identification rate was close to 100% for each subject under each stimulation condition. No reaction times were recorded in this control experiment.

### Stimulation Locations

**Figure 2:**
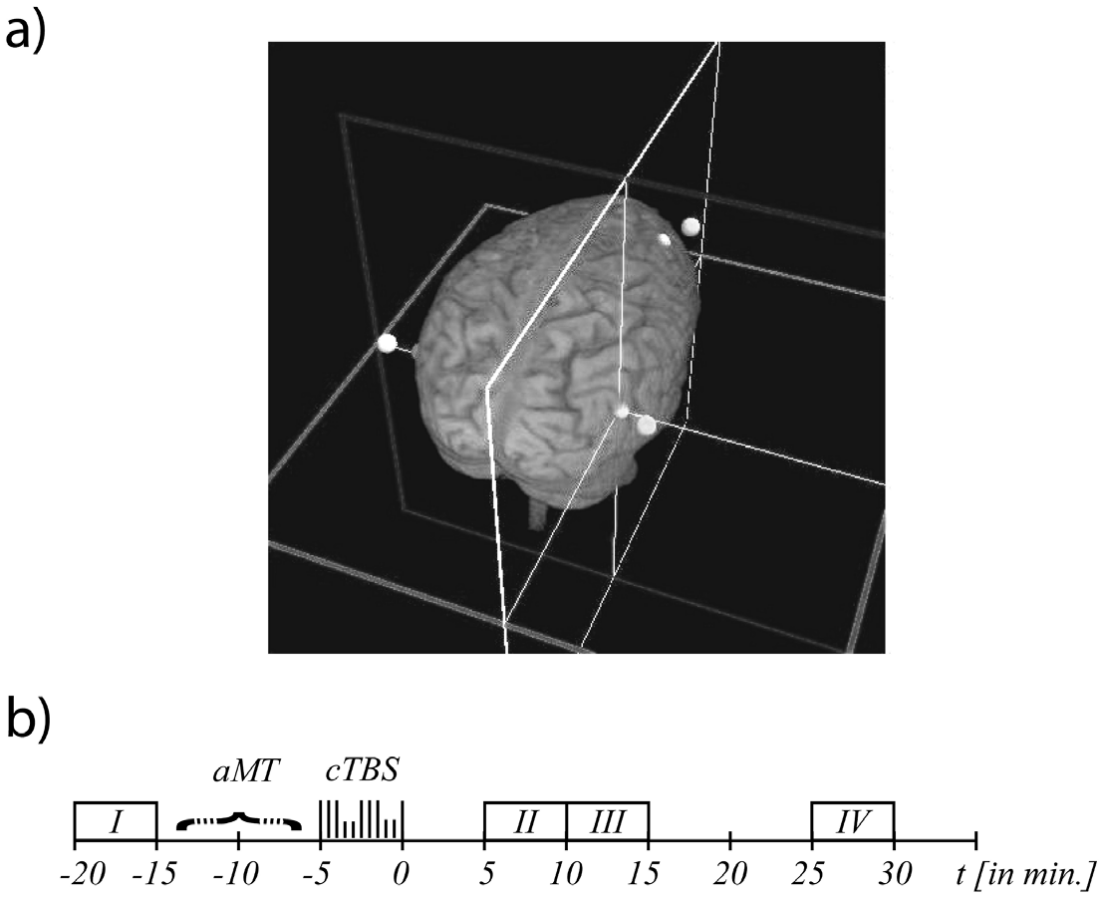
Location and design of TMS application. (a) Screenshot from the Localite Navigation System, illustrating all TMS application sites that were subsequently used in the study. (b) Stimulation Design. Blocks I to IV contained 96 trials each. Each block lasted for 289 sec. Total test duration per stimulation site was about 50 min. The end of the cTBS application was set as 0 min of the experimental session. Blocks I and IV were used as ‘Baseline’. Blocks II and III were the experimental blocks (‘TMS’). ‘aMT’, time the active Motor Threshold was established.

Prior to the TMS stimulation, T1 weighted anatomical MRI scans (176 slices of 1 mm with 256×256 voxels of 1 mm×1 mm size, TR = 2300 ms, TE = 2.92 ms, TI = 1100 ms) were acquired for all subjects in a 3T Siemens Trio Scanner (Siemens, Germany). Stimulation sites were localized by using the frameless, stereotaxic Localite Navigation System (Localite, Germany, cf. Fig. 2a). These locations included the left and right junction between the temporal, occipital and parietal lobe (TPJ; cf. Fig. 2a) and were identified according to previously observed activation sites in healthy subjects [40] as well as in a patient with simultanagnosia [39]. In detail, we ‘skullstripped’ the individual MRI image using the Localite software and identified the anatomical stimulation sites on the dorsolateral aspect of the hemisphere for every subject by anatomical landmarks. Further, corresponding entry points were defined on the dorsolateral surface to minimize the distance between TMS coil and target location.

We either stimulated the left or right TPJ alone or applied bilateral stimulation simultaneously. In addition, the right prefrontal cortex (PFC) − an area highly unlikely to be involved in any global or local object perception processes − served as a control stimulation site (cf. Fig. 2a). The PFC location was individually located so that the entry way of the TMS pulse was not reaching a gyrus but rather a sulcus [51], [52]. Each subject thus took part in four different experimental sessions (left TPJ, right TPJ, bilateral TPJ, and right PFC). Between individual sessions a resting period of at least 7 days was introduced to exclude any long lasting effect on the subjects behavior. The order of the experimental sessions was balanced over all subjects.

### Transcranial Magnetic Stimulation

Two Magstim-Super-Rapid-2 Stimulators (Magstim, UK; biphasic) with a maximal stimulator output of 1.2 T were used to deliver TMS via a figure-of-eight coil (70 mm diameter). For each subject pulse strength was set at 80% in relation to his/her active motor threshold (aMT). For all stimulation sites the coil was placed tangentially to the scalp with the handle pointing back and 45° down. The aMT was determined visually before each experimental session and was reached when a finger twitch could be elicited in 5 of 10 applications of a single pulse over the motor cortex area of the left hand (‘hand knob’). The subject held up the left arm and hand, lightly touching middle finger and thumb. The threshold was minimized by a hotspot search; the area around the hand knob was stimulated until a minimal stimulator output was found to elicit the finger twitch. The average aMT over all fifteen subjects was 55.3% of the maximal stimulator output (MSO), ranging from 41.5% to 61.5% (average male subjects: 56.9%; female: 54.4%).

Each session started with a baseline block of 96 trials without TMS stimulation (Fig. 2b) after which the aMT was established. This procedure allowed to rule out the possibility of interference between the baseline block and the determination of the aMT. Subsequently, cTBS was applied. A total number of 300 pulses was delivered for each stimulation site in 100 triple-pulses at a 5-Hz frequency, while the triple-pulses had a frequency of 50 Hz. Stimulation thus lasted for 20 seconds in total (Fig. 2b). This ‘repetitive transcranial magnetic stimulation’ protocol is also known as ‘continuous theta-burst stimulation (cTBS300)’ from previous studies [47], [53]. According to Huang et al. [47], [53] the maximal effect of the cTBS300 protocol in motor evoked potentials was observed between 7 to 14 min, while Di Lazzaro et al. [54] reported a peak interval between 5 to 10 min after the TMS application and a return to baseline after 20 minutes. Therefore, the first experimental block was initiated 5 min after the last cTBS pulse and was immediately followed by a third experimental block (Fig. 2b). The maximal effect was thus covered by two experimental blocks. After a break of 10 min a fourth block of 96 trials followed beginning 25 min after the TMS application which served as a second baseline block.

## Results

**Figure 3:**
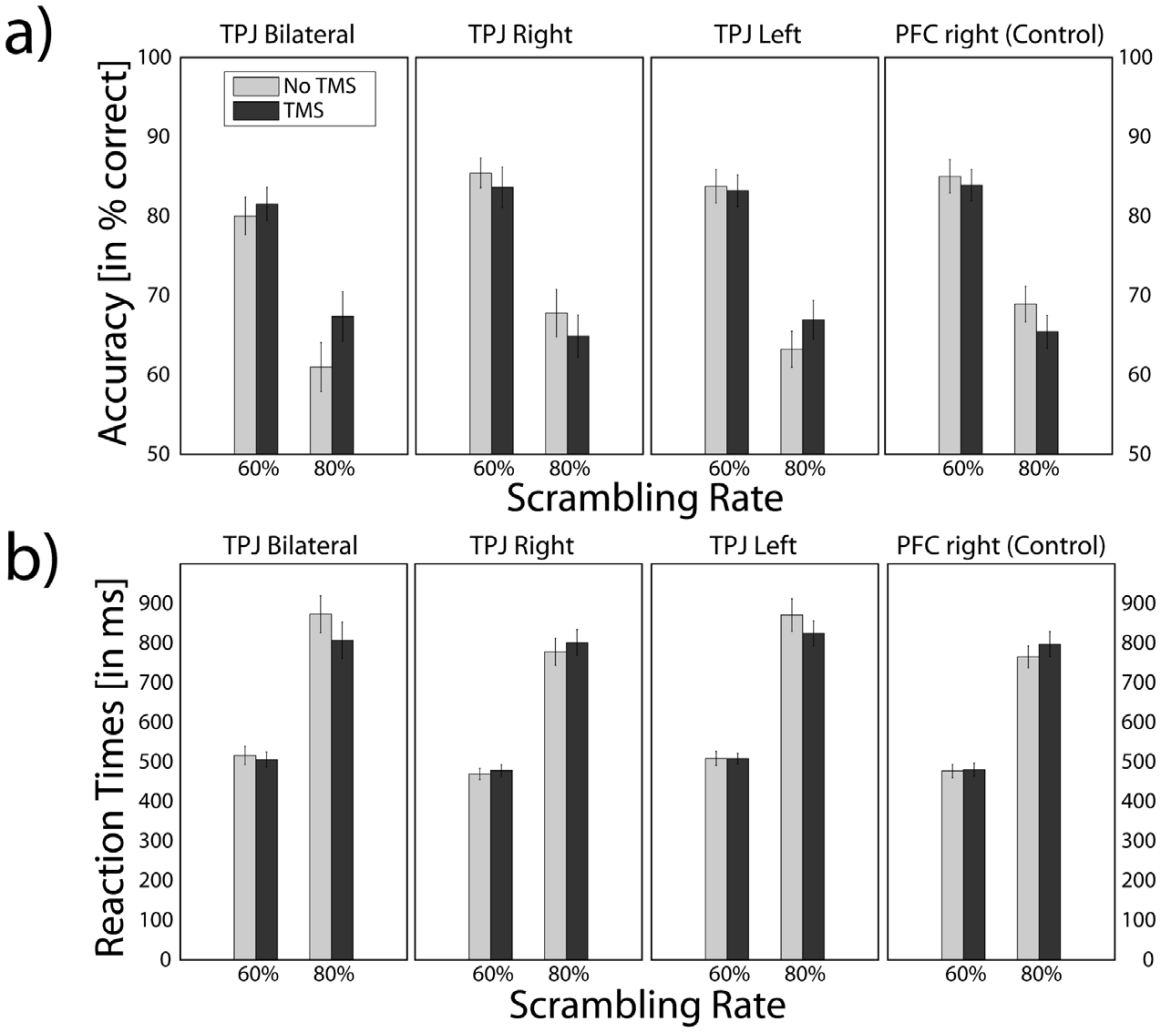
Bilateral simultaneous versus unilateral TMS application. Performance accuracy (a) and reaction times (b) for identifying the global level of the stimuli. Bright Bars represent the mean of the ‘Baseline’ (blocks I and IV); dark bars the mean of ‘TMS’ (blocks II and III). Error bars are standard errors.

Identification performance was at ceiling, i.e. close to perfect recognition, for 20% and 40% scrambling rate stimuli, with an average performance of 96.2% correct across all blocks in the conditions with and those without TMS application respectively. The 20% and 40% scrambling rate conditions thus were discarded from further analysis. For the remaining 60% and 80% scrambling rate conditions, the comparison between block I and IV without TMS (cf. Fig. 2b) revealed no significant differences neither for accuracy (paired t-tests; all experimental conditions p>0.134) nor for reaction time (all p>0.132). Therefore, these blocks were analyzed together as ‘Baseline’. In parallel, blocks II and III with TMS application were combined as ‘TMS’. Figure 3 presents the subjects’ mean accuracy and reaction times in identifying the global level of the 60% and 80% scrambling rate stimuli for ‘Baseline’ and ‘TMS’.

### Performance Accuracy

A repeated measures 4×2×2 ANOVA with Site (bilateral TPJ, left TPJ, right TPJ, control PFC), Stimulation (TMS, Baseline) and Scrambling Rate (60%, 80%) as independent factors was performed using Greenhouse-Geisser corrections to control for sphericity. The analysis revealed significant effects for Scrambling Rate (F_1,34.4_ = 678.42, p<0.001) as well as for the interaction between Site and Stimulation (F_1.7,34.4_ = 4.17, p = 0.033). Subsequent Bonferroni corrected paired t-tests for the four stimulation sites pooled for scrambling rate revealed a significantly increased accuracy following bilateral TMS application over TPJ (t = −3.53, p = 0.001), while there was no significant difference between baseline and experimental conditions for the remaining stimulated locations (Right TPJ: p = 0.184; Left TPJ: p = 0.266; Right PFC: p = 0.116).

### Reaction Times

A repeated measures 4x2x2 ANOVA with factors Site, Stimulation, and Scrambling Rate was performed using Greenhouse-Geisser corrections. This analysis revealed significant effects for Scrambling Rate (F_1,32.2_ = 725.04, p<0.001) as well as for the interaction between Site and Stimulation (F_2.5,32.2_ = 3.15, p = 0.045). Subsequent Bonferroni corrected paired t-tests for the four stimulation sites pooled for scrambling rate revealed significantly lower reaction times following bilateral TMS application over TPJ (t = 3.019, p = 0.005), while there was no significant difference between baseline and experimental conditions for the remaining stimulated locations (Right TPJ: p = 0.314; Left TPJ: p = 0.161; Right PFC: p = 0.187).

## Discussion

The present study aimed to investigate the relevance of TPJ for global Gestalt perception in humans by raising the question whether TMS application over TPJ can modulate global Gestalt perception. The findings are important for mechanisms of global Gestalt perception in general and simultanagnosia in particular. In fact, manipulation of cortical activity over TPJ resulted in behavioral changes of global Gestalt perception in healthy humans. However, a significant influence of TMS was only observed for bilateral TPJ stimulation. Unilateral stimulation had no effect on the subjects’ performance, neither in accuracy nor in reaction times. The findings are in line with the majority of patient studies, where bilateral rather than unilateral lesions of TPJ lead to simultanagnosia [22]–[31], [35], [39]. Our results thus strengthen the idea that global Gestalt perception is co-dependent on both hemispheres with a critical role of TPJ.

However, contrary to our expectation, bilateral TMS application over TPJ did not lead to decreased global Gestalt perception but rather the opposite: accuracy improved significantly and reaction times were significantly lower. These results were rather unexpected because cTBS over the motor cortex had been shown to suppress (and not increase) MEPs [47], [53], [54]. Suppression aftereffects had bewen observed for approximately 20 minutes, with peak suppression between 5 and 14 minutes (5 to 10 minutes: Di Lazzaro et al. [54]; 7 to 14 minutes: Huang et al. [47]). Targeting specifically the excitatory motor pathway which is involved in MEP generation likely explains this finding [53], [55]. However, small changes in the stimulation protocol for the motor cortex can modify cortical mechanisms and psychophysical behavior significantly. Introducing breaks of 10 seconds after 5 seconds of triple pulses (a protocol known as ‘intermediate TBS’) or breaks of 8 seconds after 2 seconds of triple pulses (‘intermittent TBS’) either had no effect or evoked facilitation (instead of suppression) of MEPs [47], [56]. Thus, it has been suggested that cTBS stimulation induces both excitatory and inhibitory effects [47], [57] with the predominance of one or the other, depending on the details of the stimulation protocol. Huang et al. [58] turned this idea into a mathematical model and proposed that TMS generally builds up excitatory and suppressing effects with the excitatory effect to be faster than the inhibitory effect. The facilitation effect saturates faster at a lower level, while the inhibition effect builds up slower and saturates at a higher level. However, Gamboa et al. [59] have shown that the predictions from this model can not be reliable. They applied cTBS over the motor cortex with protocols up to 1200 pulses, i.e. two and four times as long as the protocols used by Huang et al. [47], [53]. The initial inhibition effect with 300 and 600 pulses was now found to switch to an excitatory effect. This finding suggests that cTBS over the same simulation site can result in excitation or inhibition, depending of the length of stimulation. A similar observation was reported using these protocols (cTBS with 300 or 600 pulses [60]). Excitation or suppression was found depending on the preceding isometric voluntary muscle contraction and the number of pulses.

Beyond an influence of the stimulation protocol it is likely that also the morphology of the stimulated cortical area has a significant impact on the type of behavioural consequences (decrease vs. increase) evoked by TMS (for review [61]). The present study applied cTBS over TPJ, an area that so far has not been investigated intensively with TMS. The polarity and/or the temporal dynamics of the complex summation effects evoked by cTBS application over TPJ might be different from those evoked over the motor cortex [61]. Also, differences in long distance effects of TMS on several interconnected regions [57], [61], [62] might contribute to different behavioral effects resulting from stimulation at different cortical sites. For these various reasons a TMS stimulation protocol that induces suppression over the motor cortex might result in excitation over TPJ (e.g., TMS over TPJ might influence onging processes at other anatomical sites, thus facilitating Gestalt perception). The present observation of an increase in performance following the bilateral stimulation of the TPJ thus might have been surprising but – regarding the different effects of TMS application on cortical activity – in the end not necessarily unexpected.

If bilateral TPJ involvement is important for global Gestalt perception, why is TMS application over one hemisphere alone not sufficient to evoke a similar effect? We can only speculate about an answer. It is possible that unilateral stimulation is effective but not sufficient to induce measurable behavioral changes for the majority of the TMS stimulation protocols. If we assume that both TPJ areas influence each other by inter-hemispherical connections, bilateral stimulation might then be enough to reach a certain level of activation or deactivation. Interestingly, a recent study reported a measurable effect also by unilateral TMS stimulation of the right parietal cortex [63], suggesting that with a suitable combination of frequency, number of pulses and length of stimulation even unilateral TMS stimulation might be sufficient to induce behavioral changes. A further possibility to explain the present observation of bilateral versus unilateral stimulation effects is that indirect, long distance effects in both hemispheres leading to affection of further anatomical structures beyond area TPJ are required [57], [61]. Moreover, it remains to be investigated by future studies whether bilateral TMS stimulation, specifically with theta-burst TMS, in contrast to unilateral TMS stimulation may improve performance regardless of the specific stimulation site as well as may have a measurable effect on reaction times when subjects correctly identify objects at the local level.

In summary, our study demonstrates bilateral involvement of the temporo-parieto-occipital junction in global Gestalt perception of hierarchically organized stimuli. In line with the vast majority of observations in patients with simultanagnosia, unilateral TMS stimulation of only the right or the left TPJ was not sufficient to evoke behavioral changes in grouping local elements into a holistic percept. The present work does not allow to conclude whether excitatory or inhibitory effects alone or the combination of both in a complex network process underlies the bilateral involvement.
